# Safety considerations for ultrasound-guided nerve blocks in the emergency department: complications and best practices

**DOI:** 10.1007/s11739-026-04378-y

**Published:** 2026-05-11

**Authors:** David Martin, Joseph Stegeman, Andrew Goldsmith, Joseph Brown, Arun Nagdev

**Affiliations:** 1 Department of Emergency Medicine, Highland Hospital, Alameda Health System, Oakland, CA USA; 2 Department of Emergency Medicine, University of California San Francisco, San Francisco, CA USA; 3 Department of Emergency Medicine, Lahey Hospital, Burlington, MA USA; 4 University of Massachusetts School of Medicine, Worcester, MA USA; 5 Department of Emergency Medicine, University of Colorado-Anschutz Medical Campus, Aurora, CO USA

**Keywords:** Ultrasound-guided nerve block, Emergency medicine, Regional analgesia

Ultrasound-guided nerve blocks (UGNBs) have transformed pain management in the emergency department (ED), providing rapid, targeted analgesia for traumatic and non-traumatic conditions. The American College of Emergency Physicians recognizes UGNBs as a core procedural skill, reflecting expanding indications and strong supporting evidence [1–3]. As their utilization increases, a clear understanding of procedural safety is essential to minimize risk and promote effective implementation.

Safety principles in emergency medicine draw from decades of perioperative regional anesthesia research demonstrating a remarkably low incidence of major complications [4]. Recent registry data from 11 U.S. EDs including 2,735 patients reported a complication rate of just 0.4%, underscoring the strong safety profile of emergency clinician-performed nerve blocks [5]. Although adverse events are rare, those that do occur—such as peripheral nerve injury (PNI) and local anesthetic systemic toxicity (LAST)—necessitate clear understanding and thoughtful prevention strategies.

Safe performance of UGNBs begins with pre-procedure planning. This includes confirming the indication, reviewing relevant anatomy, performing a baseline neurologic examination, and selecting an appropriate local anesthetic based on desired onset and duration. Weight-based dosing must be calculated in advance to avoid exceeding recommended maximums, particularly when multiple blocks or large volumes are anticipated. Absolute contraindications include infection at the injection site and allergy to the anesthetic. Anticoagulation is a relative contraindication depending on block location, particularly in non-compressible areas near vessels where inadvertent vascular puncture could lead to significant bleeding [6]. A procedural time-out should confirm the patient’s identity, weight, laterality, planned dose, and availability of lipid emulsion.

Continuous needle-tip visualization is the primary safeguard against mechanical nerve injury and intravascular injection. Incremental, low-pressure injection, with intermittent aspiration, further reduces risk. Inject normal saline once the needle is in position to hydrodissect open the fascial plane and create an anechoic window for improved needle visualization. Use of echogenic block needles also improves needle visibility. Blunt-tipped needles may reduce the risk of fascicular penetration and nerve injury. Maintaining standardized block carts with block supplies, dosing guidelines, and intralipid facilitates safe implementation of UGNBs.

Post-procedure monitoring for local anesthetic systemic toxicity should continue for 30–60 min. Although high-quality evidence is limited, expert consensus recommends cardiac monitoring for large-volume fascial plane blocks or injections above the knee or elbow, where systemic absorption risk is higher [7–10]. Nursing education should define expected block effects, timing of reassessment, and escalation pathways for suspected LAST.

PNI is defined as a new or worsening sensory, motor, or autonomic deficit attributable to the block [11]. Although rare [4], injury may result from mechanical trauma, ischemic injury, or chemical neurotoxicity. Mechanical injury occurs with needle contact or penetration of the perineurium. Intrafascicular injection can elevate endoneurial pressures and compromise microvascular flow, causing ischemia [11]. Chemical toxicity reflects the dose-dependent neurotoxic potential of local anesthetics, especially when high concentrations enter the fascicle [11]. These mechanisms highlight the importance of avoiding intrafascicular injection through continuous needle-tip visualization and precise identification of the epineurium and surrounding fascial planes. In practice, the needle should be advanced into the fascial plane that surrounds the nerve—not into the nerve itself—and small amounts of saline or local anesthetic should be injected to gently open this plane. Prevention relies on slow, low-pressure injection and immediate cessation if paresthesia or pain occurs. UGNBs are generally avoided in patients with baseline neurologic deficits, as distinguishing preexisting findings from post-block complications can be challenging.

LAST is the most serious UGNB complication, though its overall incidence remains low [12]. It typically results from inadvertent intravascular injection or excessive anesthetic dosing [13]. Early symptoms include perioral numbness, metallic taste, auditory changes, agitation, or tremors, which may progress to seizures [14]. Inadvertent intravascular injection or rapid administration into highly vascular regions may precipitate early cardiovascular toxicity—such as tachycardia, hypertension, or wide-complex dysrhythmias—sometimes preceding neurologic symptoms [14]. Prevention relies on incremental low-volume injection with intermittent aspiration and continuous needle-tip visualization, combined with adherence to weight-based dosing limits and selection of shorter-acting agents when clinically appropriate. Adding epinephrine to the anesthetic may reduce systemic absorption. For larger-volume truncal blocks (≥ 20 mL), such as the serratus anterior plane or erector spinae plane blocks, reducing the total anesthetic load to no more than 50% of the maximum dose may enhance safety because of increased vascular absorption in truncal regions [15].

Treatment of LAST follows the American Society of Regional Anesthesia and Pain Medicine guidelines. Any potential LAST symptom should trigger immediate cessation of local anesthetic administration and administration of 20% lipid emulsion. Treat seizures with benzodiazepines [16]. Initial lipid bolus dosing is 1.5 mL/kg followed by an infusion of 0.25 mL/kg/min, with repeat boluses for persistent instability and a maximum cumulative dose of 10 mL/kg over 30 min. Vasopressin, local anesthetic-class antiarrhythmics, beta-blockers, and calcium-channel blockers should be avoided because of the potential to worsen local anesthetic toxicity [17].

Concern that UGNBs mask acute compartment syndrome (ACS) remains largely theoretical and based on reports of “silent” ACS in which regional analgesia obscured escalating pain [18]. Evidence suggests that regional analgesia alone is unlikely to delay diagnosis of ACS, as key findings—pain with passive range of motion, tense compartments, progressive neurologic deficits, and vascular changes—typically remain evident despite sensory blockade [19–21]. In high-risk patients, analgesia decisions should involve the consulting service. When used, single-injection UGNBs with short-acting agents and frequent reassessments may facilitate safe monitoring for evolving ACS.

Safe integration of UGNBs requires standardized departmental protocols and adherence to core safety principles. Contemporary ED data confirm a very low complication rate, aligning with long-standing regional anesthesia experience [5]. With careful technique and appropriate patient selection, UGNBs offer effective, opioid-sparing analgesia.
